# Polyunsaturated Fatty Acid Biosynthesis Across Three Trophic Levels in Freshwater Aquaculture: Current Knowledge and Perspectives

**DOI:** 10.3390/ijms27073319

**Published:** 2026-04-07

**Authors:** Evangelia Ivanova, Ivayla Dincheva, Ilian Badjakov, Vasil Georgiev

**Affiliations:** 1 Department of Agrobiotechnologies, Agrobioinstitute, Agricultural Academy, 8 Dr. Tsankov Blvd., 1164 Sofia, Bulgaria; evangelia.ivanova@abi.bg (E.I.); ivadincheva@abi.bg (I.D.); 2 Laboratory of Cell Biosystems, Institute of Microbiology, Bulgarian Academy of Sciences, 139 Ruski Blvd., 4000 Plovdiv, Bulgaria; vasgeorgiev@microbio.bas.bg

**Keywords:** PUFA, microalgae metabolism, zooplankton bioconversion, fish larvae, fatty acid desaturases, trophic transfer, lipid biosynthesis, multi-omics

## Abstract

Polyunsaturated fatty acids (PUFAs), especially the long-chain omega-3 fatty acids eicosapentaenoic acid (EPA) and docosahexaenoic acid (DHA), are essential nutrients for aquatic organisms and play key roles in growth, reproduction, neural development, and immune function. In freshwater ecosystems and aquaculture systems, the availability of these lipids depends on complex interactions within aquatic food webs, where PUFAs are produced by primary producers and transferred to higher trophic levels. This review summarizes current knowledge on the biosynthesis, regulation, and trophic transfer of PUFAs in freshwater aquaculture food webs, with particular emphasis on interactions among microalgae, zooplankton, and fish larvae. The main biochemical pathways and regulatory mechanisms responsible for PUFA synthesis in microalgae are described, together with the environmental factors that influence their production. The role of zooplankton at an intermediate trophic level is discussed, highlighting their ability to retain, modify, and transfer dietary fatty acids to higher consumers. Finally, the capacity of freshwater fish larvae to synthesize and regulate long-chain PUFAs through key metabolic enzymes is examined, along with the influence of diet and environmental conditions on these processes. By integrating information from molecular, biochemical, physiological, and ecological studies, this review provides an overview of the mechanisms underlying PUFA production and trophic transfer in freshwater aquaculture food webs.

## 1. Introduction

Polyunsaturated fatty acids are long-chain fatty acids characterized by the presence of two or more double bonds in their hydrocarbon chain [1]. They are primarily classified into two families—omega-3 (ω-3) and omega-6 (ω-6)—determined by the position of the first double bond relative to the terminal methyl end [2]. All vertebrates, including humans and fish, lack the Δ12 and Δ15 desaturase enzymes required to synthesize essential fatty acids like linoleic and α-linolenic acid *de novo*, which are the primary precursors for longer-chain omega-3 and omega-6 polyunsaturated fatty acids [3,4]. While vertebrates cannot synthesize these precursors from scratch, they possess specific elongase and desaturase enzymes that allow them to convert dietary precursors into longer-chain polyunsaturated fatty acids (LCPUFAs), though this biosynthetic capacity varies significantly by species and environment [4,5]. Biochemically, LC-PUFAs such as eicosapentaenoic acid (EPA, 20:5ω3) and docosahexaenoic acid (DHA, 22:6ω3) are integral components of cell membranes [6,7]. By incorporating into phospholipids, they regulate membrane fluidity, selective permeability, and the function of membrane-bound proteins [8]. Furthermore, LC-PUFAs serve as critical precursors for eicosanoids, highly bioactive signaling molecules like prostaglandins and leukotrienes that modulate inflammation, immune responses, and vascular physiology [2].

The transfer of these essential nutrients in aquatic environments is typically organized within a three-trophic level framework [9]. At the base of the food web, microalgae are the primary producers and synthesize PUFAs and LC-PUFAs through photosynthesis-linked metabolic pathways [10,11]. These fatty acids are then consumed and selectively retained by intermediate consumers, specifically zooplankton, which act as a vital trophic link [12]. Finally, freshwater fish occupy the consumer level, accumulating these lipids from their diet to support somatic growth, reproduction, and the development of high-density neural tissues like the brain and retina [2]. These molecules are transferred through the food web via “dietary routing”, where consumers incorporate fatty acids into their tissues with minimal modification [13].

A major challenge in modern freshwater aquaculture is maintaining high LC-PUFA levels in farmed fish [14]. By 2018, it was established that the aquaculture industry had expanded by over 6% annually over the preceding 15 years, reaching a point where it supplied more than 50% of global seafood [4]. Historically, aquaculture feeds relied on finite marine resources such as fishmeal and fish oil to provide preformed EPA and DHA [15]. However, the rapid expansion of the industry has forced a shift toward more sustainable, terrestrial plant-based ingredients [8]. While these plant oils are often rich in C18 precursors like α-linolenic acid (ALA), they are generally devoid of the long-chain ω-3 fatty acids found in marine sources [16,17]. Because the endogenous capacity of many fish to bioconvert these precursors into EPA and DHA is limited or inefficient, this dietary substitution often results in the dilution of beneficial LC-PUFAs in fish tissues [2]. Consequently, intensifying freshwater aquaculture research is essential, as many freshwater species possess a greater innate enzymatic capacity to convert C18 precursors into EPA and DHA via the Fads and Elovl pathways compared to marine species [2,4]. By leveraging metabolic traits—such as the superior innate enzymatic capacity of many freshwater species to convert C18 precursors into EPA and DHA—and through genetic selection or biotechnology to enhance this biosynthetic capacity, the aquaculture industry can reduce its dependency on finite marine fish oil resources [4]. Climate-driven alterations to marine food webs are predicted to reduce the natural availability of essential fatty acids, while the ongoing replacement of fish oil in aquafeeds has caused a decline in EPA and DHA levels, requiring consumers to potentially double their portion sizes to reach the recommended intakes [2]. Consequently, research focused on optimizing LC-PUFA biosynthetic pathways is essential to support the continued production of nutritionally high-quality aquaculture products [2].

The present review aims to summarize current knowledge on the biosynthesis, molecular regulation, and trophic transfer of PUFAs within freshwater aquaculture food webs, with particular emphasis on interactions among microalgae, zooplankton, and fish larvae. Relevant literature was identified using the Web of Science and Scopus databases. Studies published between 2003 and 2026 were considered using combinations of keywords including polyunsaturated fatty acids, LC-PUFA, EPA, DHA, microalgae, zooplankton, fish, biosynthesis, and freshwater aquaculture. The selected literature was analyzed to provide an integrated overview of PUFA production at primary producer level and their subsequent transfer and metabolic regulation across higher trophic levels in freshwater aquaculture systems.

## 2. Molecular Mechanisms of PUFA Biosynthesis in Microalgae

Microalgae are a highly diverse group of unicellular photosynthetic organisms inhabiting both marine and freshwater environments, where they form the foundation of aquatic food webs and contribute significantly to global primary production [18]. Metabolically, they are characterized by efficient carbon fixation through photosynthesis, supported by carbon-concentrating mechanisms, and many species exhibit metabolic flexibility by switching between autotrophic, heterotrophic, and mixotrophic modes depending on environmental conditions [19,20]. Importantly, microalgae possess the complete enzymatic machinery, including desaturases and elongases, required for the *de novo* synthesis of essential PUFAs, such as EPA and DHA [21]. Furthermore, they are highly efficient at assimilating inorganic nutrients like NO_3_^−^ and PO_4_^3−^ to build proteins and nucleic acids, making them robust systems for both industrial production and bioremediation [22]. Their rapid growth and biochemical diversity have also enabled the large-scale cultivation of selected species (e.g., *Chlorella*, *Nannochloropsis*) in photobioreactors and open-pond systems for aquaculture and biotechnology applications [20].

The primary substrate for fatty acid synthesis in microalgae is acetyl-CoA, which is generated through the oxidative decarboxylation of pyruvate derived from photosynthetic carbon fixation or carbohydrate breakdown [18]. This acetyl-CoA undergoes an essential carboxylation step, catalyzed by acetyl-CoA carboxylase (ACC/ACCase), to form malonyl-CoA [18]. This reaction requires ATP and biotin as cofactors [23]. Subsequently, the malonyl group is transferred from malonyl-CoA to an acyl carrier protein (ACP) by malonyl-CoA:ACP transacylase, forming malonyl-ACP [24]. Malonyl-ACP then serves as the essential two-carbon donor for the subsequent iterative elongation cycles orchestrated by the Type-II fatty acid synthase (FAS) system in the chloroplast [25]. This process culminates in the production of saturated fatty acids (SFAs), specifically palmitic acid (C16:0), which is synthesized via β-ketoacyl-ACP synthase I (KAS I), and stearic acid (C18:0), produced through further elongation by β-ketoacyl-ACP synthase II (KAS II) [26]. These early steps provide the foundational saturated fatty acids that are then modified into polyunsaturated forms [26]. In microalgae, PUFA synthesis occurs through two fundamentally different pathways, specifically the aerobic pathway involving sequential desaturation and elongation, and the anaerobic pathway mediated by polyketide synthase system [24].

### 2.1. The Aerobic Pathway

**Figure 1:**
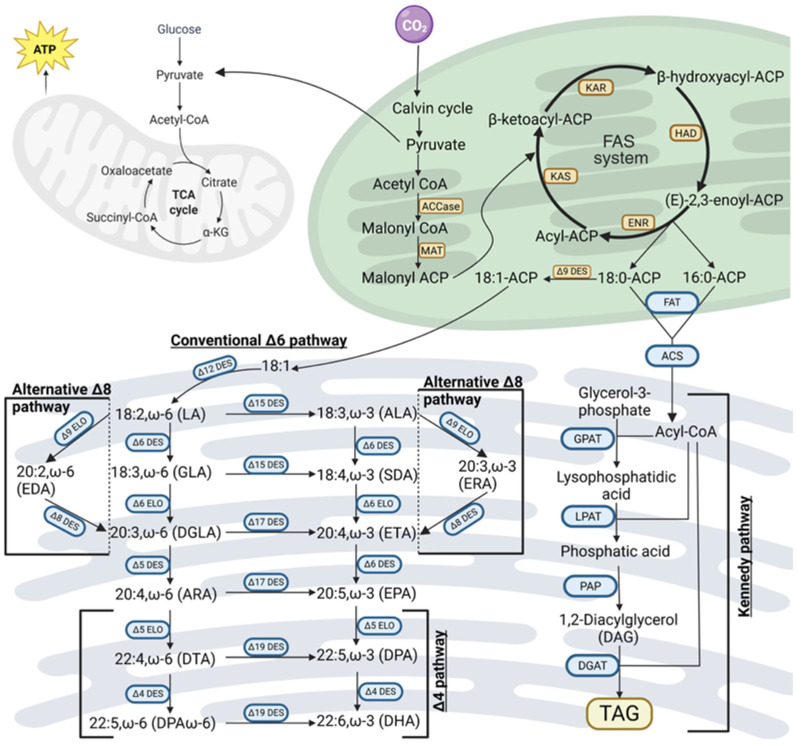
Schematic overview of the aerobic biosynthetic pathway of LC-PUFA in microalgae. Schematic representation of carbon flux from CO_2_ fixation and central metabolism toward fatty acid synthesis, LC-PUFA formation, and TAG accumulation in microalgae under aerobic conditions. Carbon fixed through the Calvin cycle or derived from carbohydrate catabolism is converted to pyruvate and subsequently to acetyl-CoA. In the chloroplast, acetyl-CoA is carboxylated to malonyl-CoA and enters the fatty acid synthase (FAS) complex, where iterative condensation, reduction, and dehydration reactions generate C16 and C18 acyl-ACP intermediates. These fatty acids are released and converted to acyl-CoA before exporting to the endoplasmic reticulum, where sequential elongation and desaturation reactions produce long-chain polyunsaturated fatty acids via the conventional Δ6 or alternative Δ8 pathways. Fatty acids are finally incorporated into triacylglycerols (TAGs) through the Kennedy pathway for storage. Abbreviations: ACCase, acetyl-CoA carboxylase; ACS, acyl-CoA synthetase; ALA, α-linolenic acid; ARA, arachidonic acid; DGLA, dihomo-γ-linolenic acid; DHA, docosahexaenoic acid; DPA, docosapentaenoic acid (ω-3); DPAn-6, docosapentaenoic acid (ω-6); DTA, docosatetraenoic acid; EDA, eicosadienoic acid; ELO, elongase; ENR, enoyl-ACP reductase; EPA, eicosapentaenoic acid; ERA, eicosatrienoic acid; ETA, eicosatetraenoic acid; FAT, acyl-ACP thioesterase; FAS, fatty acid synthase; GLA, γ-linolenic acid; GPAT, glycerol-3-phosphate acyltransferase; HAD, β-hydroxyacyl-ACP dehydratase; KAR, β-ketoacyl-ACP reductase; KAS, β-ketoacyl-ACP synthase; LA, linoleic acid; LPAT, lysophosphatidic acid acyltransferase; MAT, malonyl-CoA:ACP transacylase; PAP, phosphatidic acid phosphatase; SDA, stearidonic acid; TAG, triacylglycerol. Created in BioRender. https://BioRender.com/1dcs80m (accessed on 31 March 2026).

In the dominant aerobic pathway, these saturated precursors undergo sequential desaturation and elongation to form LC-PUFAs, as illustrated in Figure 1 [10]. The process typically begins with the introduction of a double bond at the Δ9 position of stearic acid (18:0) by stearoyl-ACP desaturase (SAD) or stearoyl-CoA desaturase (SCD) to produce oleic acid (18:1n-9) [27]. A fundamental distinction between microalgal and vertebrate metabolism is the presence of methyl-end desaturases, specifically Δ12 and Δ15 (also known as ω3) desaturases, in microalgae [19]. These enzymes enable the conversion of oleic acid into linoleic acid (LA, 18:2n-6) and further into ALA (18:3n-3) [8]. Because vertebrates lack these specific enzymes, 18:2n-6 and 18:3n-3 are essential dietary requirements for most animals [28]. Following the synthesis of these precursors, front-end desaturases (FADs)—such as Δ6\Δ5 and Δ4—and elongases (ELOVL) work coordinatively to add double bonds and extend the carbon chain [8,15]. For instance, the conventional Δ6 pathway converts ALA to stearidonic acid (SDA), which is then elongated and further desaturated to EPA (20:5n-3) and DHA (22:6n-3) [29].

### 2.2. The Anaerobic PKS Pathway

**Figure 2:**
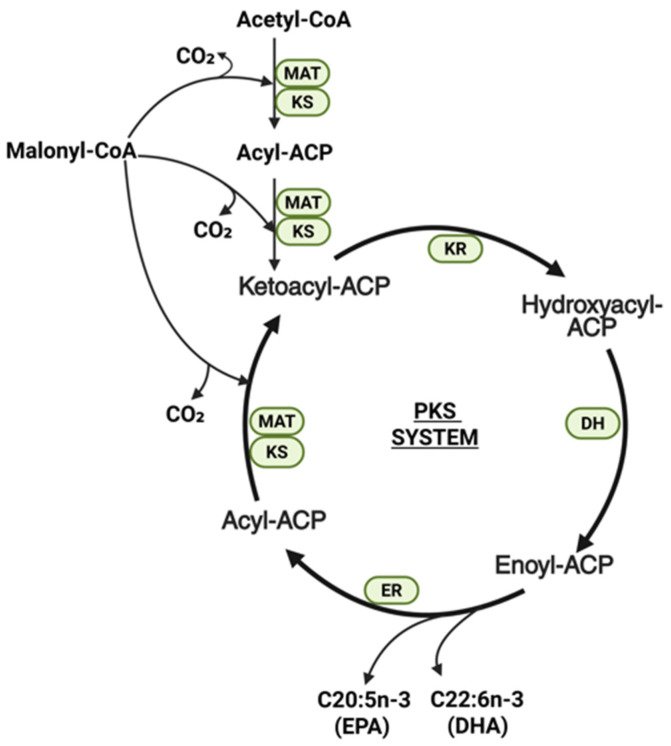
Schematic representation of the anaerobic polyketide synthase (PKS) pathway for LC-PUFA biosynthesis in microalgae. Acetyl-CoA and malonyl-CoA are sequentially condensed by the multifunctional PKS complex containing the KS, KR, DH and ER domains, thereby eliminating the need for separate desaturases and molecular oxygen and directly producing. Double bonds are introduced during chain elongation, eliminating the requirement for separate desaturases and molecular oxygen, leading directly to the production of EPA and DHA. Abbreviations: ACP, acyl carrier protein; DH, dehydratase; EPA, eicosapentaenoic acid; ER, enoyl reductase; KR, β-ketoacyl reductase; KS, β-ketoacyl synthase; MAT, malonyl-CoA:ACP transacylase; PKS, polyketide synthase. Created in BioRender. https://BioRender.com/msbj46g (accessed on 31 March 2026).

Certain marine microalgae and microorganisms, such as *Schizochytrium* and *Thraustochytrium*, employ an alternative anaerobic polyketide synthase (PKS) system, often referred to as the PUFA synthase pathway, as schematically represented in Figure 2 [30]. Unlike the aerobic pathway, this system is oxygen-independent and more energetically efficient as it requires less NADPH to produce LC-PUFAs [31]. The PKS complex is a large multifunctional enzyme containing domains such as 3-ketoacyl synthase (KS), 3-ketoacyl reductase (KR), dehydratase (DH), and enoyl reductase (ER) [30,32]. This pathway facilitates the *de novo* synthesis of DHA and EPA directly from malonyl-CoA by inserting double bonds into the growing acyl chain during the elongation process, effectively bypassing the need for separate desaturases and molecular oxygen [29].

### 2.3. Molecular Regulation

The regulation of lipid genes in microalgae is highly responsive to abiotic stressors through complex transcriptional and signaling networks [10]. Transcriptional regulation in microalgae is primarily orchestrated by several major transcription factor (TF) families, including basic leucine zipper (bZIP), MYB, basic helix-loop-helix (bHLH), and DNA binding with one finger (Dof) proteins [21,25]. These TFs act as essential metabolic sensors and regulators that bind to specific cis-acting elements in the promoters of target genes to modulate lipid metabolic pathways [27]. For example, in *Chlamydomonas reinhardtii*, the bZIP2 transcription factor is positively correlated with diacylglycerol acyltransferase during nitrogen deprivation, facilitating the accumulation of triacylglycerols (TAGs) [25]. Similarly, the MYB1 transcription factor in *Chlamydomonas* serves as a key positive regulator; its expression is significantly upregulated under nitrogen starvation, and its absence can lead to a 60% reduction in TAG accumulation due to the downregulation of Kennedy pathway genes such as LPAT, PAP, and DGAT [32].

Environmental stressors such as temperature fluctuations and nutrient availability further refine this regulatory landscape through specific induction mechanisms. In response to cold stress, microalgae employ “homeoviscous adaptation”, increasing membrane PUFA content to maintain fluidity [7]. This process is driven by the transcriptional induction of desaturase genes, such as *FAD7*, *FAD2*, and *FAB2*, which are often co-expressed with specific cold-responsive MYB-like TFs [33]. Conversely, high-temperature stress increases cellular membrane fluidity, potentially disrupting lipid bilayers [34]. To adapt microalgae remodel membranes by increasing saturated fatty acids (SFAs) and monounsaturated fatty acids (MUFAs) and decreasing PUFAs [10]. High temperature generally suppresses the expression and activity of fatty acid desaturases [35]. For example, in the cyanobacterium *Synechocystis* sp., several genes encoding desaturases are temperature-regulated, with specific ω3 desaturases being downregulated as temperatures increase, which reduces the degree of membrane unsaturation [35]. Under nitrogen or phosphorus starvation, microalgae typically redirect carbon flux away from growth and protein synthesis toward storage lipid accumulation [36,37]. In the diatom *Phaeodactylum tricornutum*, the MYB-type transcription factor PtPSR is strongly upregulated under phosphorus limitation and positively regulates phospholipid degradation genes including glycerol phosphodiester phosphodiesterase and phosphoethanolamine phosphatase. This regulatory activity drives phospholipid remodeling and promotes neutral lipid accumulation, whereas PtPSR mutants exhibit excessive phospholipid accumulation due to repression of these degradation pathways [27,32].

Epigenetic mechanisms, which involve heritable yet reversible variations in gene expression without altering the underlying DNA sequence, play a crucial role in microalgal adaptation and metabolic plasticity. These mechanisms are vital for regulating biosynthetic activity in response to environmental stressors and can influence the accumulation of valuable compounds, including lipids [20,38]. For instance, epigenetic suppression and transcriptional inactivation of foreign genes can significantly hamper stable gene expression in microalgae, posing challenges for genetic engineering efforts [20,39]. Gene silencing, a form of RNA-based regulation involving sequence-specific RNA targeting and degradation, has been observed in various algal species, with some cases linked to high-level methylation of foreign genes [20]. This phenomenon also impacts the stability of transgenes in engineered microalgae, where factors such as non-coding RNAs, promoters, and even high GC content can affect gene silencing [40].

### 2.4. PUFA Profiles

The fatty acid (FA) and lipid composition of microalgae varies dramatically, with taxonomy serving as the dominant factor explaining this variation, often superseding environmental conditions [1,6]. Different microalgal classes possess distinct biochemical signatures. Freshwater diatoms (Bacillariophyceae) and cryptophytes (Cryptophyceae) are generally rich in EPA and/or DHA and excellent quality food resources for zooplankton [6]. Chlorophytes typically contain high levels of short-chain PUFAs like ALA but lack or only have traces of EPA and DHA [12]. Cyanobacteria generally provide low dietary omega 3 PUFAs, but some also contain higher levels of ALA and LIN [6,12]. Chlorophytes and Cyanobacteria are of very poor food quality for zooplankton [6], as summarized in Table 1, which compares the nutritional quality of freshwater microalgae species. Marine microalgae, in particular, produce higher amounts of PUFAs than freshwater species, which is crucial for survival in marine environments [30]. While phylogeny is the strongest driver, environmental factors (e.g., temperature, light, and nutrient limitation) further modify lipid composition within species by altering the proportions of SFA, MUFA, and PUFA to maintain membrane fluidity [10,41]. 

This variability in microalgal FA profiles directly dictates the nutritional landscape for zooplankton, which serve as the vital link transferring energy and essential biomolecules to higher trophic levels [6,45]. Because most zooplankton lack the enzymatic capacity to synthesize essential PUFA *de novo*, their somatic growth, reproduction, and survival are intrinsically constrained by the biochemical quality of their primary producer diet [12]. Consequently, the specific FA signatures identified in the phytoplankton community define the nutritional value available to consumers, with those rich in long-chain PUFAs like EPA and DHA acting as high-quality resources compared to the nutritionally deficient profiles of species like cyanobacteria [12]. Once ingested, this dietary input is processed via complex physiological mechanisms such as selective retention and metabolic bioconversion, which enable zooplankton to maintain essential biomolecule levels despite environmental fluctuations [12]. This transition from producer-derived biochemical diversity to consumer-level physiological requirements underscores the fundamental role of zooplankton in navigating and regulating their dietary environment to satisfy specific metabolic needs [12].

## 3. Molecular Bioconversion and Selective Retention in Zooplankton

**Figure 3:**
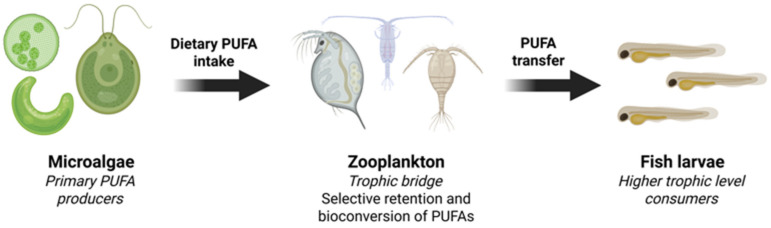
Trophic transfer of polyunsaturated fatty acids through the aquatic food web. Created in BioRender. https://BioRender.com/bwt8u3c (accessed on 31 March 2026).

Freshwater zooplankton comprise a diverse group of primary and secondary consumers occupying intermediate trophic levels, including rotifers, copepods, cladocerans, protozoans, and ostracods [46]. They serve as key intermediaries in aquatic food webs, facilitating the transfer of energy and essential biomolecules from primary producers to higher trophic levels [47]. Because zooplankton rely on microalgae as their primary food source, the population health and nutritional success of these consumers are fundamentally linked to the biochemical composition of available phytoplankton [9,46]. Thus, the targeted mass cultivation of nutrient-rich microalgae is critical in aquaculture to support zooplankton populations, which serve as essential live feeds for larval fish and crustaceans [48]. As primary consumers, zooplankton act as a critical trophic bridge, actively processing, bioconverting, and selectively retaining dietary fatty acids to meet the physiological requirements rather than functioning as passive conduits [12]. The trophic transfer of PUFAs is illustrated in Figure 3. Consequently, the targeted cultivation of PUFA-rich microalgae is essential in aquaculture systems to support zooplankton populations used as live feed for larval fish and crustaceans [48].

### 3.1. Intermediate Processing of Dietary Fatty Acids

The processing of dietary lipids varies significantly across zooplankton taxa, reflecting differences in metabolism and life history traits [12]. Cladocerans, such as *Daphnia*, exhibit rapid lipid turnover; experimental evidence shows that *Daphnia magna* can replace more than 50% of their total fatty acids within only two days of a diet switch. By the sixth day of exposure to a new diet, they typically reach stable total fatty acid (TFA) and phospholipid fatty acid (PLFA) profiles [49]. While zooplankton primarily derives lipids from their food, they possess a vital ability to bioconvert C18 precursors into long-chain (LC) PUFAs [49]. Results from δ^13^C signatures of individual fatty acids provide evidence that *Daphnia* synthesize EPA (20:5n-3) via the elongation and desaturation of ALA (18:3n-3) or SDA (18:4n-3) [49]. However, the efficiency of this conversion can be low, such as a measured ALA to EPA conversion efficiency of only 0.5% in the ubiquitous freshwater grazer *Daphnia* sp. [1]. Taxon-specific patterns are also evident in the field: cladocerans predominantly focus on EPA retention, whereas copepods maintain high levels of DHA (22:6n-3) for membrane structure regardless of dietary variability [12].

### 3.2. Transcriptome Patterns in Response to PUFA Availability

Dietary PUFA availability serves as a master regulator of gene expression related to growth and immunity in zooplankton. In *Daphnia magna*, the supply of EPA directly influences the eicosanoid pathway, which is essential for signaling, reproduction, and immune function [50,51]. Specific genes involved in oogenesis, such as vitellogenin (VTG1), are significantly upregulated when EPA is available in the diet, suggesting that EPA acts as a biochemical trigger for egg production [50].

The transcriptional response of eicosanoid-related genes is strongly implicated in immune defense in *Daphnia* [51]. When *Daphnia magna* raised on a C20-PUFA-free diet based on the green alga *Chlamydomonas globosa* are challenged with the bacterium *Pasteuria ramosa*, the COX-like chorion peroxidase Pxt is strongly induced, and the intracellular phospholipase *A*_2_ gene *iPlA*_2_ is specifically upregulated under these PUFA-deficient conditions [51]. This suggests that in the absence of dietary LC-PUFA, *Daphnia* mobilize eicosanoid precursors from membrane phospholipids via PLA_2_ activity to sustain prostanoid production during immune activation [51]. Dietary C20-PUFAs also modify basal gene expression: provision of ARA or EPA reduces Cox transcript levels while strongly increasing *Pxt* expression, supporting PXT as an important prostanoid-producing enzyme when external PUFA substrates are available [51]. Adequate dietary ARA and EPA can also improve fecundity and performance under pathogen challenge, indicating that PUFA availability may buffer the trade-off between reproduction and immunity [51]. These nutritional effects interact with temperature through homeoviscous adaptation, as PUFA requirements increase at lower temperatures to maintain membrane fluidity [50]. Consistent with this, EPA enhances the expression of *Pxt* and the reproduction-related gene *VTG1* in *D. magna*, illustrating how dietary PUFA availability and thermal regime jointly modulate eicosanoid- and reproduction-related transcriptional responses [50].

### 3.3. Selective Allocation and Molecular Buffering

Zooplankton exhibit a molecular “buffering” capacity, allowing them to maintain optimal levels of essential PUFAs even when dietary quality is poor [12]. Experimental diet-switching has shown that *Daphnia* switched from a high-quality to a moderate-quality diet preferentially retain the most physiologically important FAs, specifically EPA and ARA, in their membranes for up to 14 days [49]. This buffering functions as a metabolic relay, where zooplankton acts as a homeostatic filter, dampening the environmental volatility transmitted from fluctuating primary producers [12].

This regulatory capacity is reflected in the multivariate analysis of seston–zooplankton interfaces, where consumer fatty acid “ellipses” are significantly smaller than those of their diet, indicating that internal regulation dampens the impact of environmental variation [12]. While microalgae biochemical profiles fluctuate wildly in response to nutrient or temperature stress, zooplankton selectively sequester EPA and ARA into polar lipid fractions to preserve biochemical integrity [12,49]. Because egg production represents a major drain on a female’s lipid reserves, *Daphnia* prioritizes the allocation of EPA into eggs, maintaining higher concentrations in the offspring than in the somatic tissue of the mother [52]. This selective retention ensures that even under fluctuating seston quality, zooplankton can sustain essential physiological processes and provide a high-quality nutritional base for upper trophic level consumers [49]. Through this homeostatic relay, zooplankton effectively stabilize the biochemical energy flow, mitigating the impacts of primary producer instability and supporting the survival, growth, and neurological development of sensitive fish larvae [47,53].

## 4. Metabolic Pathways and Genetic Control in Freshwater Fish

The capacity for LC-PUFA biosynthesis in fish is significantly influenced by habitat and evolutionary history [54]. Freshwater and anadromous species, such as common carp (*Cyprinus carpio*) and salmonids like rainbow trout (*Oncorhynchus mykiss*), generally possess a more complete molecular machinery to convert C18 precursors into physiologically vital C20 and C22 LC-PUFAs compared to marine teleosts [2]. This diversity in biosynthetic capability is an evolutionary adaptation to the availability of fatty acids in different environments; marine phytoplankton are naturally rich in EPA and DHA, whereas freshwater food webs are characterized by higher levels of C18 precursors like ALA and LA but lower levels of DHA [55]. Consequently, freshwater fish have maintained the functional genes *FADs2* and *ELOVL5* required to endogenously produce essential LC-PUFAs from dietary 18-carbon precursors [2,56]. In contrast, many marine species have lost or exhibit very low expression of these genes because their natural diets are high in preformed LC-PUFA [8]. Ultimately, this metabolic continuum—originating from LC-PUFA production in microalgae and their trophic transfer through zooplankton—determines the availability of essential fatty acids for fish, particularly EPA and DHA, which are critical for neural development and survival in larval stages [2].

### 4.1. Rate-Limiting Enzymes and Multifunctional FADs2

The fatty acid desaturase 2 (*FADs2*) gene has been identified as a critical rate-limiting enzyme and the primary bottleneck in the LC-PUFA biosynthetic pathway [57]. Unlike mammals, the vast majority of teleosts have lost the *FADs1* gene (Δ5 desaturase), which has driven a remarkable functional diversification of *FADs2* [54]. In freshwater species, *FADs2* often exhibits multifunctional desaturase activities, including Δ6, Δ5, and Δ8 capabilities [54]. For example, in the common carp and ayu sweetfish, *FADs2* possesses the Δ6 activity required to initiate the pathway from C18 PUFAs and the Δ5 activity necessary to complete the synthesis of EPA and ARA [54,58]. This multifunctionality allows these fish to bypass the enzymatic constraints associated with the loss of *FADs1* [54]. Furthermore, while the conversion from EPA to DHA typically follows the Sprecher pathway involving two elongations and a second Δ6 desaturation, the efficiency of this process in fish remains heavily dependent on the substrate preference and expression levels of *FADs2* and elongases like *ELOVL5* [59,60].

### 4.2. Transcriptional Regulation and Nuclear Receptors

The expression of endogenous LC-PUFA biosynthetic genes is highly flexible and regulated by dietary lipid sources [61]. High dietary levels of LC-PUFAs, particularly docosahexaenoic acid (DHA), exert a potent feedback inhibition on the biosynthetic pathway [4]. It is now established that DHA, rather than EPA, acts as the primary metabolic signal that suppresses the expression of key genes such as Δ6 *FADs2* [4]. Conversely, when fish are fed vegetable oil (VO) diets rich in C18 precursors (e.g., linoleic and α-linolenic acids) but devoid of LC-PUFAs, this transcriptional suppression is relieved, leading to the typically observed upregulation of biosynthetic genes as a physiological adaptation to maintain membrane homeostasis [62]. In species like common carp and Chinese sturgeon, hepatic and brain mRNA levels of *Δ6-FAD*, *ELOVL5*, and *ELOVL2* are significantly higher on VO-based diets compared to those fed fish oil diets [59,62].

This transcriptional regulation is mediated by dietary fatty acids acting as ligands for specific transcription factors and nuclear receptors [14]. Sterol regulatory element-binding proteins (SREBPs), notably *SREBP1*, are recognized as key regulators of both lipogenesis and LC-PUFA biosynthesis in teleosts [4,63]. The upregulation of desaturases and elongases on VO diets is largely attributed to relieving the suppression normally exerted by dietary LC-PUFAs via *SREBP-1* [63]. However, the regulatory role of SREBPs can be complex; for instance, in common carp, hepatic *SREBP1* was found to be upregulated in fish fed diets enriched with EPA and DHA, suggesting that its activity may also depend on reaching species-specific optimal nutritional requirements [63].

While peroxisome proliferator-activated receptors (PPARs), particularly *PPARα*, are activated by PUFAs and modulate genes involved in fatty acid oxidation and lipid homeostasis, their role in coordinating the upregulation of the biosynthetic pathway is less consistent [4,64]. In sterlet sturgeon eggs, a VO diet rich in C18 precursors actually resulted in a significant decrease in *PPARα* mRNA levels, suggesting that its expression may be more closely tied to the specific ratio of n-3/n-6 PUFAs or available energy levels rather than acting as a universal trigger for endogenous synthesis [64]. Similarly, in pikeperch larvae, dietary DHA and ARA levels significantly influenced *PPARα* expression but failed to influence the mRNA levels of Δ6 desaturase or *ELOVL5* [65]. These findings indicate that while PPARs are sensitive to dietary LC-PUFAs, they may regulate other physiological responses—such as immune function, stress tolerance, and eicosanoid metabolism—rather than directly driving the core biosynthetic genes in all species or life stages [65].

## 5. Multi-Omics Approaches to Elucidate PUFA Metabolism in Aquatic Systems

Traditional biochemical approaches can describe FA composition but provide limited insight into the regulatory mechanisms controlling PUFA biosynthesis and trophic transfer [17,58]. The integration of genomics, transcriptomics, proteomics, and metabolomics enables a system-level understanding of nutrient–gene interactions and metabolic regulation [32]. By linking genetic potential, gene expression, protein abundance, and metabolite profiles, multi-omics approaches identify key enzymes, regulatory pathways, and metabolic nodes involved in PUFA production [31].

Genomic and transcriptomic analyses have been central to identifying fatty acyl desaturases and elongases, which catalyze sequential desaturation and elongation steps in PUFA biosynthesis [8,66]. Comparative genomics have revealed interspecific variation in the complement of these genes, reflecting differences in metabolic capacity and evolutionary adaptation [8]. Many freshwater fish species retain the complete enzymatic pathway to synthesize LC-PUFAs from C18 precursors, whereas many marine species rely more heavily on dietary LC-PUFAs [8]. Gene copy number variation also contributes to metabolic capacity; freshwater populations of three-spined sticklebacks exhibit increased *FADs2* copy number associated with enhanced DHA synthesis [67,68].

Transcriptomics provides a dynamic view of PUFA pathway regulation by revealing condition-dependent gene expression [25]. In fish, diets rich in vegetable oils commonly induce the upregulation of desaturase and elongase genes as a compensatory response to low dietary LC-PUFAs [4]. Temperature also modulates expression, with cold stress increasing desaturase transcription to maintain membrane fluidity [25]. In common carp, transcriptomic comparisons between high- and low-PUFA phenotypes revealed the differential expression of genes involved in oxidation and ubiquitination, suggesting that regulatory and turnover processes influence the final PUFA levels [58]. However, transcript abundance does not always correspond to protein levels or enzyme activity because of post-transcriptional and post-translational regulation [25,40].

In microalgae, integrated omics datasets have resolved the three-step architecture of PUFA biosynthesis: acetyl-CoA supply, elongation/desaturation through fatty acid synthase FAS–elongase–desaturase or polyketide synthase (PKS) routes, and triacylglycerol (TAG) assembly [32]. Multi-omics screening has identified alternative LC-PUFA pathways and regulatory nodes, including acetyl-CoA carboxylase (ACC), diacylglycerol acyltransferase (DGAT), and NADPH-generating reactions, whose modulation increases EPA and DHA accumulation [25,32]. Transcriptomic analyses in Nannochloropsis gaditana demonstrated that light quality altered desaturase gene expression and EPA levels [31]. In *Chlorella sorokiniana* used in wastewater treatment, exposure to free nitrous acid increased the expression of genes involved in fatty acid and TAG synthesis, resulting in elevated lipid accumulation [22]. Proteomic analyses in *Phaeodactylum tricornutum* under dark stress or nutrient deprivation identified changes in lipid biosynthesis enzymes such as pyruvate dehydrogenase, enoyl-ACP reductase, stearoyl-ACP desaturase, and TAG lipase, providing functional confirmation of pathway remodeling [25].

In zooplankton, genomic discoveries have shown that several invertebrate groups possess methyl-end desaturases enabling *de novo* PUFA synthesis, revising assumptions about strict dietary dependence in aquatic food webs [8]. For example, in the marine copepod *Tigriopus californicus*, genomic evidence indicates a complete DHA biosynthetic pathway [8]. Transcriptomic studies in *Daphnia magna* further linked dietary EPA availability to altered expression of genes in eicosanoid signaling and vitellogenesis, connecting molecular regulation to reproductive performance [50]. These findings demonstrate how multi-omics approaches connect trophic resource quality to physiological responses.

Metabolomics provides a functional readout of metabolic state by quantifying fatty acid precursors, intermediates, and end products [19]. Using GC-MS and LC-MS platforms, metabolomic profiling validates whether transcriptional changes translate into altered biochemical composition [31]. Integrated transcriptomic–metabolomic analyses have identified metabolic bottlenecks and regulatory nodes that are not apparent from single-layer analyses [19]. When combined with compound-specific stable isotope analysis (CSIA), metabolomics enables the tracing of carbon flow and distinguishing between direct dietary routing and endogenous bioconversion of PUFAs [53]. CSIA demonstrated that some freshwater zooplankton obtain PUFAs from terrestrial pollen inputs [1]. In carp, CSIA revealed that while bulk lipids originated from cereal-based feeds, essential LC-PUFAs were derived from natural prey, and isotopic signatures provided evidence of endogenous bioconversion [53].

Proteomics complements transcriptomics by confirming the abundance of enzymes and identifying post-translational modifications regulating activity [25]. It reveals shifts between lipid biosynthesis and β-oxidation under environmental stress, providing mechanistic insight into metabolic reallocation [32]. However, proteomics faces technical challenges, including protein extraction difficulties in microalgae and detection limits for low-abundance regulatory proteins [19,38].

Despite the advances of multi-omics, several limitations remain. Many datasets are fragmented and restricted to one or two omics platforms, limiting comprehensive network reconstruction [32,38]. Integration of high-dimensional datasets requires advanced bioinformatic tools and standardized analytical pipelines [38]. Moreover, laboratory-based studies may not fully capture the ecological complexity of freshwater systems, where multiple abiotic and biotic drivers simultaneously influence metabolism and trophic interactions [68]. Overall, multi-omics approaches provide an integrated framework for linking genetic capacity, regulatory dynamics, enzymatic activity, and metabolic flux to understand PUFA biosynthesis in freshwater ecosystems [19,32].

## 6. Genetic and Biotechnological Approaches for Enhancing PUFA Biosynthesis

Genetic and biotechnological strategies have emerged as powerful tools to enhance long-chain polyunsaturated fatty acid biosynthesis in freshwater aquaculture species, complementing insights gained from omics approaches [8].

Selective breeding approaches, particularly marker-assisted selection (MAS), exploit naturally occurring genetic variation associated with lipid metabolism [66]. Coding and promoter single nucleotide polymorphisms (SNPs) within *fads2* and *elovl5* gene clusters in common carp have been correlated with variation in PUFA content, including EPA, highlighting their potential as targets for genetic improvement programs [66]. Heritability studies in species such as Atlantic salmon further indicate that LC-PUFA biosynthetic capacity is partially genetically controlled, supporting the feasibility of selective breeding to enhance endogenous production [4]. Gene copy number variation (CNV), particularly in *Fads2*, has also been identified as an adaptive mechanism enhancing DHA biosynthesis in low-DHA freshwater environments, as demonstrated in sticklebacks [8].

Transgenic and genome-editing technologies enable the direct manipulation of LC-PUFA biosynthetic pathways [8]. Transgenic approaches have increased LC-PUFA production through the introduction of heterologous desaturase and elongase genes [8]. Genes derived from organisms such as masu salmon and *Caenorhabditis elegans* (*fat-1* and *fat-2*) have been expressed in model species including zebrafish, common carp, and nibe croaker, resulting in enhanced LC-PUFA biosynthesis [8]. Genome editing using CRISPR/Cas9 has validated the functional importance of key enzymes, with knockout of *elovl2* in zebrafish and Atlantic salmon leading to reduced DHA levels and impaired elongation of polyunsaturated fatty acids [8].

Nutrigenomic approaches represent an indirect strategy for modulating endogenous LC-PUFA synthesis [4]. Dietary replacement of fish oil with vegetable oils induces compensatory upregulation of *fads2* and *elovl* genes, reflecting metabolic plasticity in response to reduced dietary LC-PUFA availability [4]. This response is transcriptionally mediated, as reflected by the upregulation of *fads* and *elovl* genes under reduced dietary LC-PUFA availability, while sterol regulatory element-binding proteins (SREBPs) are recognized more broadly as key regulators of lipogenesis, cholesterol biosynthesis, and LC-PUFA biosynthesis [4].

Advances in genomics and multi-omics technologies have facilitated the identification of key enzymatic components and regulatory networks underlying LC-PUFA biosynthesis [8]. In microalgae, biotechnological improvement strategies include random mutagenesis, adaptive laboratory evolution (ALE), and targeted approaches to enhance lipid productivity and LC-PUFA production [31]. Random mutagenesis using physical and chemical methods enables the generation of large mutant libraries, with combined atmospheric and room temperature plasma (ARTP) and chemical mutagenesis achieving significant increases in EPA production [31].

Targeted genetic engineering and synthetic biology approaches provide precise control over metabolic pathways in microalgae [69]. Genome editing tools such as CRISPR/Cas9, along with advanced variants including base editing and CRISPRi/CRISPRa systems, enable targeted modification and the regulation of gene expression [19]. RNA interference (RNAi) further enables post-transcriptional gene silencing, while metabolic engineering strategies focus on the overexpression of key enzymes and disruption of competing pathways to enhance lipid accumulation [19,69]. Alternative genome editing platforms, including CRISPR/Cpf1, TALENs, and zinc finger nucleases (ZFNs), also enable targeted genome modification through site-specific DNA cleavage and repair mechanisms [39]. Integration of multi-omics datasets with computational modeling supports the identification of metabolic bottlenecks and the optimization of pathway fluxes, reducing reliance on empirical approaches [37].

## 7. Conclusions

Polyunsaturated fatty acids, particularly long-chain omega-3 fatty acids such as EPA and DHA, represent fundamental biochemical currencies that link primary producers to higher trophic levels in freshwater ecosystems. This review highlights how PUFA availability and composition are shaped by tightly regulated metabolic and genetic processes across microalgae, zooplankton, and freshwater fish, forming a dynamic trophic network that ultimately determines the nutritional quality of aquaculture products.

At the base of the food web, microalgae exhibit remarkable metabolic plasticity, governed by sophisticated transcriptional and epigenetic regulation, enabling them to modulate lipid biosynthesis in response to environmental stressors. Zooplankton acts not merely as passive vectors but as biochemical gatekeepers, selectively retaining and bioconverting essential fatty acids to stabilize PUFA transfer across fluctuating nutritional landscapes. Freshwater fish, in turn, display pronounced genetic flexibility in long-chain PUFA biosynthesis, reflecting evolutionary adaptation to freshwater environments characterized by limited preformed EPA and DHA availability.

Despite substantial advances, major knowledge gaps persist. These include limited understanding of transcriptional networks governing PUFA metabolism in natural ecosystems, incomplete characterization of species-specific bioconversion efficiencies in zooplankton, and insufficient resolution of genotype–diet interactions controlling endogenous biosynthesis in freshwater fish. Addressing these gaps will require integrative multi-omics approaches, coupled with ecological experimentation and nutritional modeling.

From an applied perspective, leveraging molecular plasticity across trophic levels offers promising avenues for enhancing the sustainability and nutritional value of freshwater aquaculture. Strategies such as selective breeding for enhanced desaturase activity, optimized feed formulations rich in metabolic precursors, and controlled manipulation of algal lipid profiles could substantially improve endogenous LC-PUFA production while reducing reliance on marine-derived feed ingredients. Ultimately, a systems-level understanding of PUFA metabolism across trophic networks will be essential for developing resilient, health-promoting, and environmentally sustainable aquaculture practices.

## Figures and Tables

**Table 1:** Nutritional quality of the freshwater microalgae classes.

Microalgae Class	Dominant FAs That Effect Food Quality	Zooplankton Food Quality Rating	Species	Ref.
Chlorophyceae	High in α-linolenic acid and oleic acid; essentially no EPA or DHA	Intermediate—provides essential ALA but lacks the most valuable long-chain PUFA (EPA/DHA)	*Chlamydomonas reinhardtii*; *Tetradesmus obliquus*	[6,42,43]
Trebouxiophyceae	Abundant ALA and oleic acid; lacks EPA/DHA	Intermediate—provides essential ALA but lacks EPA/DHA	*Chlorella vulgaris*; *Botryococcus braunii*	[6,43]
Bacillariophyceae	Rich in EPA (7–23% of total FAs) and also contains DHA; high 16:0, 14:0	Excellent—EPA/DHA make it a high-quality food source	*Cyclotella meneghiniana*; *Navicula pellicosa*	[6,42,44]
Cryptophyceae	Contains EPA, DHA and other long-chain PUFA (22:5ω6, 18:4ω3); also high ALA	Excellent—presence of EPA/DHA indicates very good food quality	*Cryptomonas ovata*; *Rhodomonas minuta*	[6,42]
Raphidophyceae	Contains EPA, DHA, and several long-chain PUFA (15:4ω3, 20:4ω3, 22:4ω6)	Excellent—EPA/DHA presence places it among the best food resources	*Gonyostomum semen*	[6]
Chrysophyceae	Contains stearidonic acid and ALA; EPA/DHA generally absent	Intermediate—essential ALA present, but EPA/DHA missing	*Dinobryon cylindricum*; *Mallomonas caudata*	[6,42]
Cyanophyceae	Contains 16:0 and 18:0; very low or absent PUFAs	Low quality or even unsuitable food for most zooplankton	*Nostoc commune var. Sphaeroides*; *Arthrospira platensis*	[6,42,43]
Euglenophyceae	Unique C15-C17 PUFA plus EPA and DHA; high ALA as well	Excellent ^1^—EPA/DHA plus a suite of unique PUFA give it top nutritional value	*Euglena gracilis*	[6,42]

^1^ The classification of intermediate food quality for classes that provide the essential short-chain PUFA ALA but lack the highly beneficial long-chain EPA/DHA, and excellent quality for classes that contain substantial EPA and/or DHA (the most critical EFAs for zooplankton growth and reproduction).

## Data Availability

No new data were created or analyzed in this study.

## Abbreviations

The following abbreviations are used in this manuscript:

PUFAs	Polyunsaturated fatty acids
LC-PUFAs	Long chain-polyunsaturated fatty acids
EPA	Eicosapentaenoic acid
DHA	Docosahexaenoic acid
ALA	α-Linolenic acid
ACP	Acyl carrier protein
Malonyl-CoA	Malonyl coenzyme A
ACC/ACCase	Acetyl-CoA carboxylase
Acetyl-CoA	Acetyl coenzyme A
Enoyl-ACP	Enoyl acyl carrier protein
KAS I	β-ketoacyl-ACP synthase I
KAS II	β-ketoacyl-ACP synthase II
SAD	Stearoyl-ACP desaturase
SCD	Stearoyl-CoA desaturase
ELOVLs	Elongases
FADs	Desaturases
SDA	Stearidonic acid
PKS	Polyketide synthase system
KS	3-ketoacyl synthase
KR	3-ketoacyl reductase
DH	Dehydratase
ER	Enoyl reductase
NADPH	Nicotinamide adenine dinucleotide phosphate
TF	Transcription factor
bZIP	Basic leucine zipper
bHLH	Basic helix-loop-helix
Dof	DNA binding one finger
TAGs	Triacylglycerols
MYB	Myb like transcription factor
LPAT	Lysophosphatidic acid acyltransferase
PAP	Phosphatidic acid phosphatase
DGAT	Diacylglycerol acyltransferase
SFAs	Saturated fatty acids
MUFAs	Monounsaturated fatty acids
PtPSR	Phosphorus starvation response transcription factor
GC content	Guanine-cytosine content
LIN	Linolenic acid
ARA	Arachidonic acid
EFAs	Essential fatty acids
TFA	Total fatty acid
PLFA	Phospholipid fatty acid
VTG	Vitellogenin
Pxt	Cyclooxygenase-like gene
iPLA_2_	Intracellular phospholipase A_2_
VO	Vegetable oil
SREBPs	Sterol regulatory element-binding proteins
PPARs	Peroxisome proliferator-activated receptors
FAS	Fatty acid synthase
GS-MS	Gas chromatography-mass spectrometry
LC-MS	Liquid chromatography-mass spectrometry
CSIA	Compound-specific stable isotope analysis
